# Toward malaria elimination in Botswana: a pilot study to improve malaria diagnosis and surveillance using mobile technology

**DOI:** 10.1186/1475-2875-11-S1-P96

**Published:** 2012-10-15

**Authors:** Simon Chihanga, Allison Tatarsky, Tjantilili Mosweunyane, Mpho Motlaleng, Lesedi Bewlay, Katy Digovich, Enele Mankhi, Deepika Kandula, Nesredin J Oumer, S Muza, A Akrem, James Makuka

**Affiliations:** 1 National Malaria Programme, Ministry of Health, Gaborone, Botswana; 2 Clinton Health Access Initiative, Boston, Massachusetts, USA; 3 Positive Innovation for the Next Generation, Gaborone, Botswana; 4 Integrated Disease Surveillance and Response, Ministry of Health, Gaborone, Botswana; 5 District Health Management Team, Ministry of Health, Chobe District, Botswana

## Background

Identifying every suspected malaria case, correctly diagnosing those cases, and reporting cases accurately in a timely matter are critical in Botswana’s move toward malaria elimination. Based on current evidence on the use of mobile phones for reporting disease incidence from health facilities in several African countries for HIV/AIDS, TB, and malaria [1-3], mobile phone-based malaria case reporting in Botswana was piloted to understand the true incidence of malaria and markedly improve surveillance and response for elimination.

## Materials and methods

The Botswana Ministry of Health conducted a pilot study in 14 health facilities in malaria-endemic Chobe District to test a Malaria Early Epidemic Detection System using GPS-enabled smartphones with customized menus to monitor, map, and trigger response to malaria cases. Nearly all health workers from the district were trained centrally and onsite to effectively build capacity on the phone-based reporting system, malaria case management, and active case detection. Activities were reinforced by regular visits by supervisors to each health facility over 15 months.

Health workers diagnosed patients using RDTs and collected blood smears and dried blood spots for malaria PCR for all RDT-positive cases. Data was entered in the smartphones per confirmed case (immediate case-based notification), weekly (aggregate data), and per case investigation. The system then sent data in real-time to a secure website developed for the pilot with automated graphical and tabular analyses. GPS coordinates were also collected from households during case investigation that were then projected onto maps on the website. Simultaneously, the system sent a weekly email to district and national officials with aggregated data for monitoring, as well as an SMS immediately to the same officials when a single case was notified through the smartphones.

## Results

The pilot clearly demonstrated the importance of parasito-logical diagnosis to reveal the true burden of malaria in a district that was assumed to have endemic malaria - the number of clinical cases reported in Chobe declined from 2,092 cases in 2010 to 164 cases in 2011 and 22 cases in 2012 through the end of the pilot in June. The proportion of suspected cases tested markedly increased from an estimated 11% to 98% during the pilot project. Just over 97% percent of all those tested were found negative, yielding a positivity rate of 2.6%. All positive cases were notified through mobile phones within 48 hours of diagnosis. An average of 77% of health facilities sent reports on key indicators through the phones each week against a set target of 80%. Select positive malaria cases were successfully mapped by residence, investigated, and monitored for 28 days to field test the new active case-based surveillance tools and system.

## Conclusion

The pilot significantly improved the accuracy, timeliness and geographic pinpointing of confirmed malaria cases, critical in an elimination programme to identify and clear transmission foci. Onsite training of health workers in case management and smartphone technology greatly improved diagnostic practices and response to positive cases. Combined, these activities will substantially increase Botswana’s ability to target and treat remaining cases, prevent onward transmission, and advance the country toward its elimination goal.
